# Immune checkpoint inhibitor therapy for ACTH-secreting pituitary carcinoma: a new emerging treatment?

**DOI:** 10.1530/EJE-20-0151

**Published:** 2020-10-05

**Authors:** Bastiaan Sol, Jeroen M K de Filette, Gil Awada, Steven Raeymaeckers, Sandrine Aspeslagh, C E Andreescu, Bart Neyns, Brigitte Velkeniers

**Affiliations:** 1 Department of Endocrinology, UZ Brussel, Laarbeeklaan, Brussels, Belgium; 2 Department of Medical Oncology, UZ Brussel, Laarbeeklaan, Brussels, Belgium; 3 Department of Radiology, UZ Brussel, Laarbeeklaan, Brussels, Belgium

## Abstract

**Background:**

Pituitary carcinomas are rare but aggressive and require maximally coordinated multimodal therapies. For refractory tumors, unresponsive to temozolomide (TMZ), therapeutic options are limited. Immune checkpoint inhibitors (ICI) may be considered for treatment as illustrated in the present case report.

**Case:**

We report a patient with ACTH-secreting pituitary carcinoma, progressive after multiple lines of therapy including chemotherapy with TMZ, who demonstrated disease stabilization by a combination of ipilimumab (anti-CTLA-4) and nivolumab (anti-PD-1) ICI therapy.

**Discussion:**

Management of pituitary carcinoma beyond TMZ remains ill-defined and relies on case reports. TMZ creates, due to hypermutation, more immunogenic tumors and subsequently potential candidates for ICI therapy. This case report adds support to the possible role of ICI in the treatment of pituitary carcinoma.

**Conclusion:**

ICI therapy could be a promising treatment option for pituitary carcinoma, considering the mechanisms of TMZ-induced hypermutation with increased immunogenicity, pituitary expression of CTLA-4 and PD-L1, and the frequent occurrence of hypophysitis as a side effect of ICI therapy.

## Background

Pituitary carcinomas are rare, accounting for 0.1% of pituitary tumors (1). Cerebrospinal and/or distant metastases are present by definition. They require maximally coordinated multimodal therapies because of their aggressive behavior (2). Therapy as proposed by the European Society of Endocrinology (ESE) includes surgical resection, adjuvant radiotherapy and first-line chemotherapy with temozolomide (TMZ) (3, 4). Nonetheless, the mortality rate of pituitary carcinoma remains high, with an average life expectancy of 2.6 years (1). Evidence regarding the next line beyond TMZ is lacking. Novel treatment modalities are urgently needed for refractory cases. Immunotherapy, with immune checkpoint inhibitors (ICI) targeting cytotoxic T-lymphocyte-associated antigen-4 (CTLA-4), programmed cell death 1 (PD-1) or its ligand (PD-L1) has been a revolution for a wide range of malignancies, with an ever-growing list of indications. In 2018, Lin *et al.* successfully treated a first case of aggressive ACTH-secreting pituitary carcinoma with ipilimumab (anti-CTLA-4) and nivolumab (anti-PD-1) combination immunotherapy (5). We now report the second case of a patient with corticotroph pituitary carcinoma with disease stabilization after the introduction of ipilimumab and nivolumab.

## Case

A 41-year-old patient was diagnosed with an invasive ACTH-secreting pituitary adenoma in 2012. Initial pathological examination had revealed positive chromogranin, p53 (1+) and strong ACTH staining. Ki-67 proliferation index was low (<1%). Initial treatment consisted of transsphenoidal and transcranial surgery with subsequently two sessions of stereotactic radiosurgery in 2013 and 2015.

**Figure 1:**
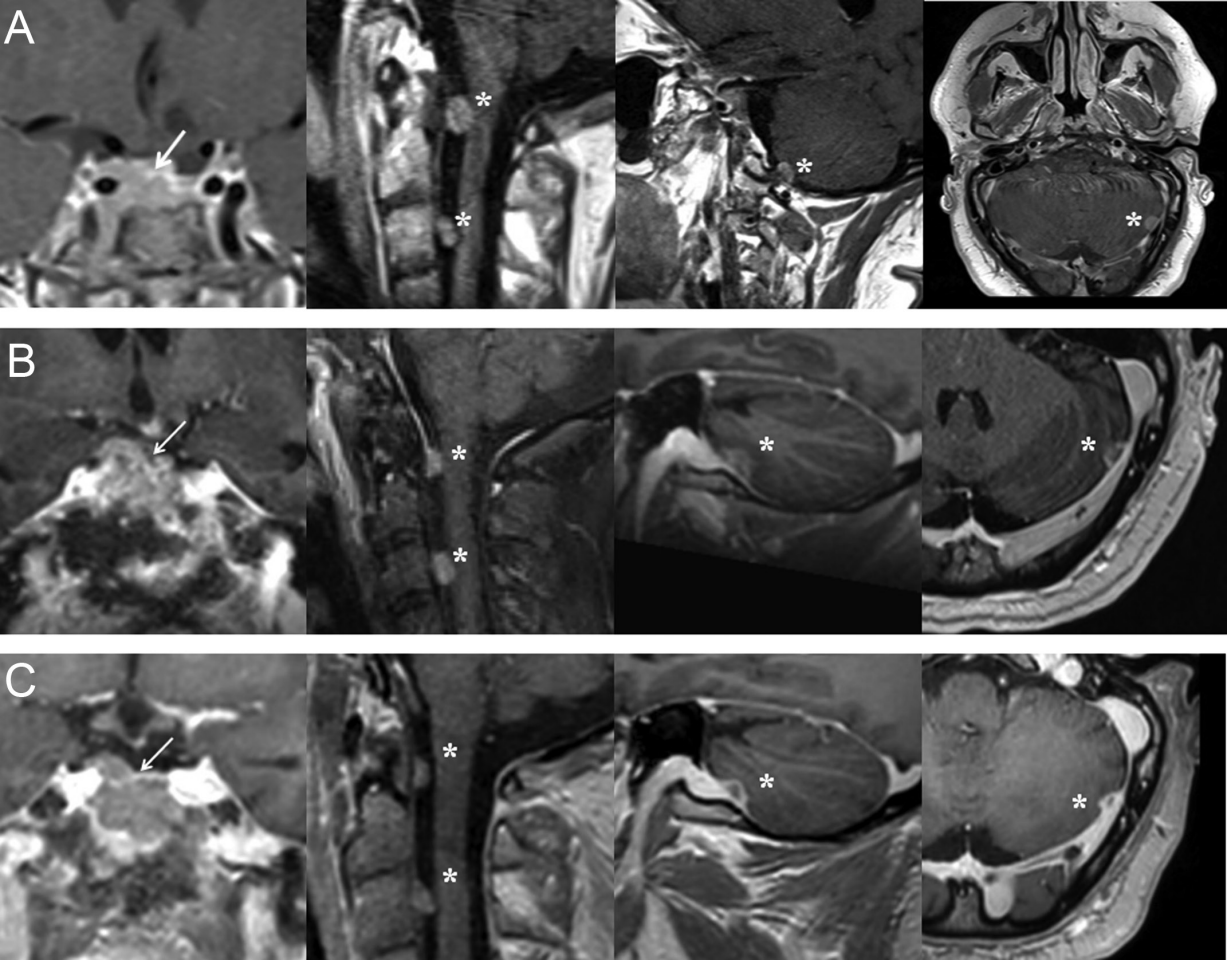
Gadolinium-enhanced T1-weighted magnetic resonance imaging of the pituitary carcinoma. Panel A shows invasion of the cavernous sinus (arrow) and the metastases of the posterior fossa, left cerebellum and cervical drop metastases at the level of the dens and the third cervical vertebra (asterix) (May 2018). Panel B shows evaluation of the pituitary carcinoma after nine cycles of TMZ treatment (April 2019). Panel C shows stable disease (irRECIST criteria) 1 year after initiation of ICI (April 2020). IrRECIST, immune-related Response Evaluation Criteria in Solid Tumors; ICI, Immune Checkpoint Inhibitors.

**Figure 2:**
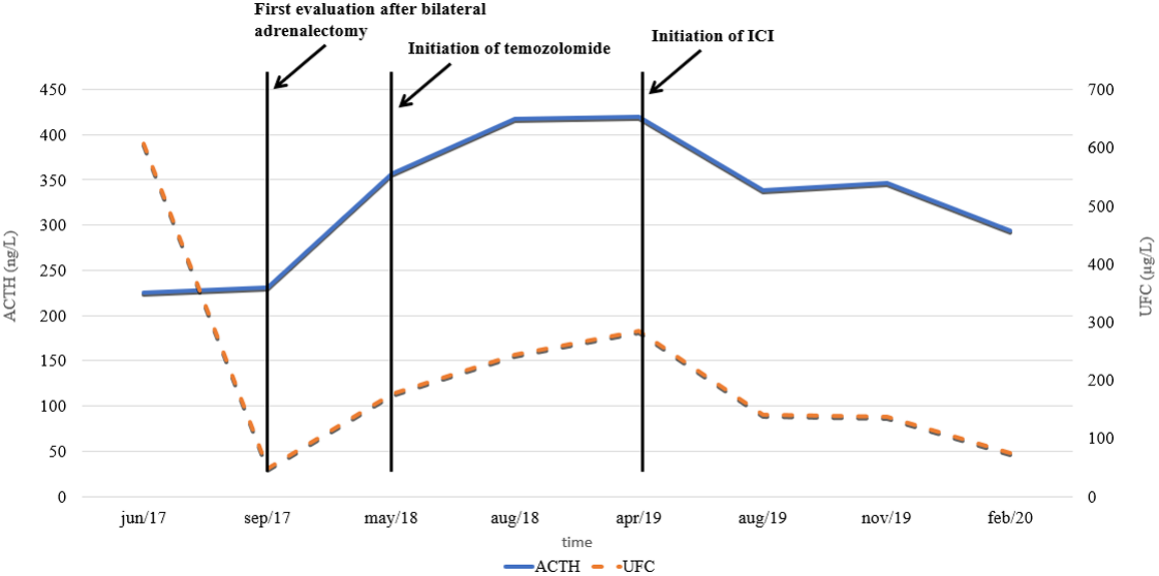
Timeline of 08:00 h ACTH and UFC during follow-up. ACTH, Adrenocorticotropic hormone (08:00 h normal range: 7.2–63 ng/L); ICI, Immune Checkpoint Inhibitors; UFC, Urinary Free Cortisol (normal range: 4.2–60 µg/24 h). A full color version of this figure is available at https://doi.org/10.1530/EJE-20-0151.

In 2017, he was first seen at our pituitary outpatient clinic for a second opinion. His therapy consisted of ketoconazole 1000 mg/day, together with thyroid and testosterone hormone replacement therapy. Hormonal workup revealed persistent Cushing’s disease (CD) with elevated 08:00 h ACTH (225 ng/L, 08:00 h normal range: 7.2–63 ng/L) and cortisol (378.5 µg/L, 08:00 h normal range: 62–180 µg/L) and high 24-h urinary free cortisol (UFC) (1727.7 µg/24 h = 606.2 µg/L, normal range: 4.2–60 µg/24 h) despite the maximal dosage of ketoconazole. MRI of the brain showed no macroscopic disease. Pasireotide 0.6 mg twice daily was initiated, but had to be discontinued for severe iatrogenic diabetes mellitus. Cabergoline 0.25 mg twice weekly was started with unsatisfactory response. Bilateral adrenalectomy was performed in September 2017. Unfortunately, follow-up revealed small residual adrenal tissue on the left side. The patient refused a second surgery for complete removal of the adrenal remnant. Disease control was nonetheless achieved with stable 08:00 h ACTH (231 ng/L), cortisol (151.6 µg/L) and UFC (126.9 µg/24 h = 47.8 µg/L). In May 2018, the patient deteriorated with the development of diplopia due to left abducens nerve palsy. Plasma ACTH had increased (357.3 ng/L) and MRI revealed recurrence of the pituitary tumor with suprasellar and cavernous sinus invasion. Additionally, metastases were detected in the posterior fossa, left cerebellum and cervical drop metastases at the level of the dens and the third cervical vertebra (Fig. 1). These findings confirmed the evolution toward corticotroph pituitary carcinoma, possibly in the context of Nelson's syndrome given the rapid progression after the bilateral adrenalectomy. Biopsy of the pituitary carcinoma for reanalysis of proliferative markers could not be performed at the time; the metastases were considered unsafe for biopsy. TMZ chemotherapy (150–200 mg/m^2^, 5 days in a 28-day cycle) was promptly initiated. First evaluation after three cycles of TMZ showed persistent CD with stable tumor burden. Ketoconazole (800 mg/day) was restarted and TMZ therapy was continued for a total of nine cycles (April 2019), when clinical progressive disease was suspected with the development of right oculomotor and abducens nerve palsies and increasing 08:00 h ACTH (419.9 ng/L) and cortisol (208 µg/L). However, no radiological progression could be detected. The patient agreed to start with a combination ICI therapy of ipilimumab (3 mg/kg) and nivolumab (1 mg/kg) every 3 weeks, for 4 cycles in a compassionate use setting (Table 1). Initial evaluation after the first four cycles demonstrated disease stabilization with declining ACTH and cortisol levels (ACTH: 338.9 ng/L; cortisol: 199 µg/L, 24-h urinary cortisol: 354.8 µg/24 h = 140.7 µg/L) without radiological change. Maintenance therapy with nivolumab (240 mg) was then continued every 2 weeks. Up to now, the patient has a non-progressive disease, one year after the initiation of ICI, with declining 08:00 h ACTH and UFC as shown in Fig. 2. Radiological disease stabilization is observed when comparing MRI obtained after the last TMZ cycle with follow-up imaging one year after the initiation of ICI (Fig. 1). However, there is no resolution of the diplopia. He did not experience any immune-related adverse events. His CD is still under control with 800 mg of ketoconazole daily.

**Table 1:** Data of plasma 08:00 h ACTH, 08:00 h cortisol, and UFC during follow up with the corresponding treatment.

Date	08:00 h Cortisol^1^ (µg/L)	08:00 h ACTH^2^ (ng/L)	UFC^3^ (µg/L)	Treatment
Jun/17	378.5	225	606.2	Ketoconazole 1000 mg + Pasireotide 0.6 mg twice daily started
Sep/17	151.6	231	47.8	Bilateral adrenalectomy already performed + hydrocortisone and fludrocortisone suppletion started
May/18	132	357.3	177	Hydrocortisone and fludrocortisone stopped + temozolomide 350 mg 5 days every 4 weeks + ketoconazole 800 mg restarted
Aug/18	145	418	243.8	Temozolomide 350 mg 5 days every 4 weeks (nine cycles) + ketoconazole 800 mg
Apr/19	208	419.9	284.7	Ipilimumab (3 mg/kg) and nivolumab (1 mg/kg) every 3 weeks + ketoconazole 800 mg
Aug/19	199	338.9	140.7	Nivolumab 240 mg every 2 weeks + ketoconazole 800 mg
Nov/19	196	346.9	136.6	Nivolumab 240 mg every 2 weeks + ketoconazole 800 mg
Feb/20	74	293.7	74.1	Nivolumab 240 mg every 2 weeks + ketoconazole 800 mg

^1^Cortisol, 08:00 h normal: 62–180 µg/L; ^2^Adrenocorticotropic hormone, 08:00 h normal range: 7.2–63 ng/L; ^3^Urinary free cortisol, normal range: 4.2–60 µg/24 h.

## Discussion

Pituitary carcinomas are rare and progress with poor survival despite maximal multimodal therapy. Pathophysiology remains poorly understood, but development in the context of Nelson’s syndrome has been reported (6). Furthermore, histopathological or immunohistochemical (IHC) analyses have not always been able to consistently predict tumor behavior. Management guidelines beyond TMZ rely on case reports. This is the second case of a patient with ACTH-secreting pituitary carcinoma, refractory to TMZ chemotherapy, treated with ICI (ipilimumab and nivolumab). Given the aggressive nature of pituitary carcinoma, the non-progressive disease with a decline in ACTH values illustrates the efficacy of the immunotherapy. The current guidelines for pituitary carcinoma recommend TMZ as a first-line chemotherapy (3). TMZ is an alkylating pro-drug that methylates DNA at the O6 position of guanine, which induces DNA damage and apoptosis. Induction of mutations in DNA mismatch repair genes causes a state of hypermutation, with the formation of novel oncogenic drivers, resulting in tumor resistance to TMZ. The same process also creates novel tumor antigens, rendering patients more immunogenic and subsequently potential candidates for ICI therapy (7).

The pituitary gland itself confers a particular immunogenicity as hypophysitis is a frequently encountered endocrine adverse event to ICI with an estimated incidence of around 10%, especially with ipilimumab. It also occurs occasionally during PD-1/PD-L1 blockade (8, 9, 10). For reasons yet unknown, hypophysitis is mainly observed in male patients (4/1 ratio) (8). Ipilimumab and tremelimumab are both CTLA-4 targeting monoclonal antibodies. CTLA-4 expression is present in normal pituitary glands and pituitary adenomas, including one autopsy case who presented a necrotizing form of tremelimumab-induced hypophysitis through type II (IgG dependent) and type IV (T-cell dependent) immune mechanisms (8). The expression of PD-L1 in pituitary adenomas has also been assessed. PD-L1 expression was found in both functioning and non-functioning pituitary tumors, with higher levels in functioning (GH- and PRL-expressing) adenomas, while tumor-infiltrating lymphocytes were also observed and correlated with PD-L1 expression (11, 12).

Considering these findings, the use of ICI therapy for the treatment of pituitary carcinoma should be considered. Lin *et al.* were the first to publish a case of a patient with an ACTH-secreting pituitary carcinoma who initially responded to TMZ and capecitabine chemotherapy, prior to metastasizing to the liver (5). They recorded a dramatic response to a combination of ipilimumab and nivolumab immunotherapy, with a reduction in tumor volume of the pituitary lesion (59%) and liver metastasis (92%) with concomitantly a severe drop in circulating ACTH levels (from 45.550 to 66 pg/mL). Genomic sequencing of the pituitary tumor (before chemotherapy) and liver metastasis (after chemotherapy) showed evidence of alkylating chemotherapy-induced somatic mutations with the presence of a *MSH6* mutation in the TMZ-treated liver metastasis. Our patient did not benefit from mutational analysis nor CTLA-4 or PD-L1 IHC, as the surgical resection of his pituitary tumor was performed at a different medical center and the remaining anatomic-pathological samples were of insufficient quality. Furthermore, the metastases were unsafe for biopsy.

Pituitary carcinomas are rare and evidence-based approach for treatment is difficult. Case reports may contribute to a better understanding of the pathophysiological mechanism involved, as well as to the possibility for personalized molecular targeted therapies. This case adds support to the possible role of ICI in the treatment of pituitary carcinoma, not responsive to the classical proposed multimodal therapy and TMZ chemotherapy. Two ongoing interventional trials, ‘DART: Dual Anti-CTLA-4 and Anti-PD-1 Blockade in Rare Tumors’ of the National Cancer Institute (NCI) and ‘Phase II Trial of Nivolumab Plus Ipilimumab in Patients With Aggressive Pituitary Tumors’ of the Memorial Sloan Kettering Cancer Center, USA, may provide further evidence for this hypothesis.

## Conclusion

Treatment options for refractory pituitary carcinoma are currently limited with poor prognosis when TMZ is ineffective. We consider ICI therapy to be a valid treatment alternative after prior TMZ therapy, considering the mechanisms of TMZ-induced hypermutation involving increased immunogenicity, the pituitary expression of CTLA-4 and PD-L1, and the frequent occurrence of hypophysitis as a side effect of ICI therapy. This is the second case of a patient with aggressive ACTH-secreting pituitary carcinoma, refractory to TMZ chemotherapy, who demonstrated benefit by a combination of ipilimumab and nivolumab checkpoint blockade therapy.

## Declaration of interest

The authors declare that there is no conflict of interest that could be perceived as prejudicing the impartiality of the research reported.

## Funding

This research did not receive any specific grant from any funding agency in the public, commercial or not-for-profit sector.

## Patient consent

Informed consent has been obtained from the patient for publication of the case report and accompanying images.
